# Efficacy and Side Effects of Green Tea Extract on Facial Skin Aging: A Systematic Review

**DOI:** 10.1111/jocd.71125

**Published:** 2026-08-28

**Authors:** Taryn Lubbers, Cyrus W. Abrahamson, Paavali A. Hannikainen, Daniel Stuart, James C. Wang

**Affiliations:** ^1^ School of Medicine Texas Tech University Health Sciences Center Lubbock Texas USA; ^2^ Feinberg School of Medicine Northwestern Medicine Chicago Illinois USA; ^3^ Division of Facial Plastic and Reconstructive Surgery, Department of Otolaryngology‐Head and Neck Surgery Northwestern Medicine Chicago Illinois USA

**Keywords:** catechin, facial aging, green tea, green tea extract, wrinkles

## Abstract

**Background:**

There has been an increasing demand for natural cosmeceuticals that can be used for their anti‐aging effects. Green tea extract (GTE) is a powerful antioxidant making it a promising candidate for combating photodamage and skin aging.

**Aims:**

To establish the effectiveness and side effects of using topical GTE on the face to improve skin appearance and photoaging.

**Methods:**

This systematic review was conducted according to PRISMA guidelines. Studies were excluded if they did not utilize the epigallocatechin gallate (EGCG) component of GTE, did not focus on facial skin aging, were not controlled trials, used a green tea beverage exclusively, or if the full text was unavailable.

**Results:**

Four hundred eighty‐one studies were identified, from which seven were extracted. Five studies measured skin elasticity which all found some degree of improvement after treatment with GTE. Four of the five studies looking at wrinkles reported a decrease in fine line wrinkles. The effect of GTE on skin irritability (erythema, inflammation, and dryness) was debated. Other notable effects include an improvement in skin tone, texture, radiance, scavenging ability, firmness and density.

**Conclusion:**

GTE shows potential as an anti‐aging compound for facial skin, but more studies are needed as the literature is varying and sparse.

**Trial Registration:**

PROSPERO: CRD42024610718

## 1 Introduction

The beauty industry has long been concerned with facial rejuvenation. This goal is achieved through many different means such as surgery, injections, and topical formulations. Recently, there has been a push from consumers to find less invasive and more natural ways to maintain a youthful appearance. Green tea extract (GTE) is one natural cosmeceutical that is currently being investigated as an anti‐aging product due to its unique properties [1].

UVB rays from sunlight are the major factor contributing to skin cancer and skin aging [2, 3]. Solar radiation is an extremely harmful carcinogen, causing most skin malignancies, which are rapidly increasing in incidence [2, 4]. UVB also contributes to the production of reactive oxygen species (ROS) and the degradation of the extracellular matrix (ECM) which both play a major role in skin aging [2, 3, 4, 5]. The creation of ROS in fibroblasts causes oxidative stress‐induced apoptosis, which makes skin wrinkle due to decreased regenerative capacity [5]. Fibroblasts that do not undergo apoptosis are irreversibly damaged and can no longer make components of the ECM: hyaluronic acid, collagen, elastin, and fibrillin [3, 5]. A depleted and nonfunctional ECM also contributes to skin wrinkling and sagging; it is usually accompanied by redness, dark spots, and inflammation [2, 5]. These factors directly contribute to the photoaging process.

GTE is made from the leaves of the *Camellia sinensis* plant that is found in Southeast Asia and China [4, 6]. For many centuries, green tea has been consumed around the world as a drink due to its many health benefits [6]. Some of these proposed benefits include antioxidant, anti‐inflammatory, chemopreventive, neuroprotective, cardioprotective, antimicrobial, and immunosuppressive effects [2, 3, 4, 5, 6, 7, 8]. These benefits are hypothesized to be due to biologically active polyphenols called catechins [6]. The four major catechins found in GTE are: epigallocatechin‐3‐gallate (EGCG), epigallocatechin (EGC), epicatechin gallate (ECG), and epicatechin (EC) [6].

EGCG is specifically being looked at as a second line antioxidant [8]. First line antioxidants are enzymes that prevent ROS creation; second line antioxidants scavenge free radicals, and third line antioxidants try to repair DNA damage [8]. Several in vitro studies have confirmed significant antioxidant and scavenging effects of EGCG in human skin cells [2, 4, 7]. EGCG inhibits hydrogen peroxide formation as well as MAPK pathways which drastically decrease oxidative stress [2, 4, 6]. A decrease in Bax protein (pro‐apoptotic) and an increase in Bcl‐2 protein (anti‐apoptotic) were also found to be side effects of EGCG use [4, 5]. EGCG has also shown to decrease levels of vascular endothelial growth factor (VEGF) and hypoxia‐inducible factor 1‐alpha (HIF‐1a) which are implicated in erythematous conditions and tumor progression due to their role in angiogenesis [4, 6].

These modifications to cellular signaling all contribute to GTE's antioxidant effects. These unique qualities are promising for using GTE as an anti‐aging cosmeceutical. While there have been several smaller studies that have investigated GTE in this capacity, there has been no systematic review that has consolidated these results to draw more unified conclusions. Therefore, the aim of this study is to review all original human studies on this topic and determine the efficacy and side effect profile of GTE, so physicians can counsel patients on its use.

## 2 Materials and Methods

### 2.1 Guidelines and PICO

**FIGURE 1:**
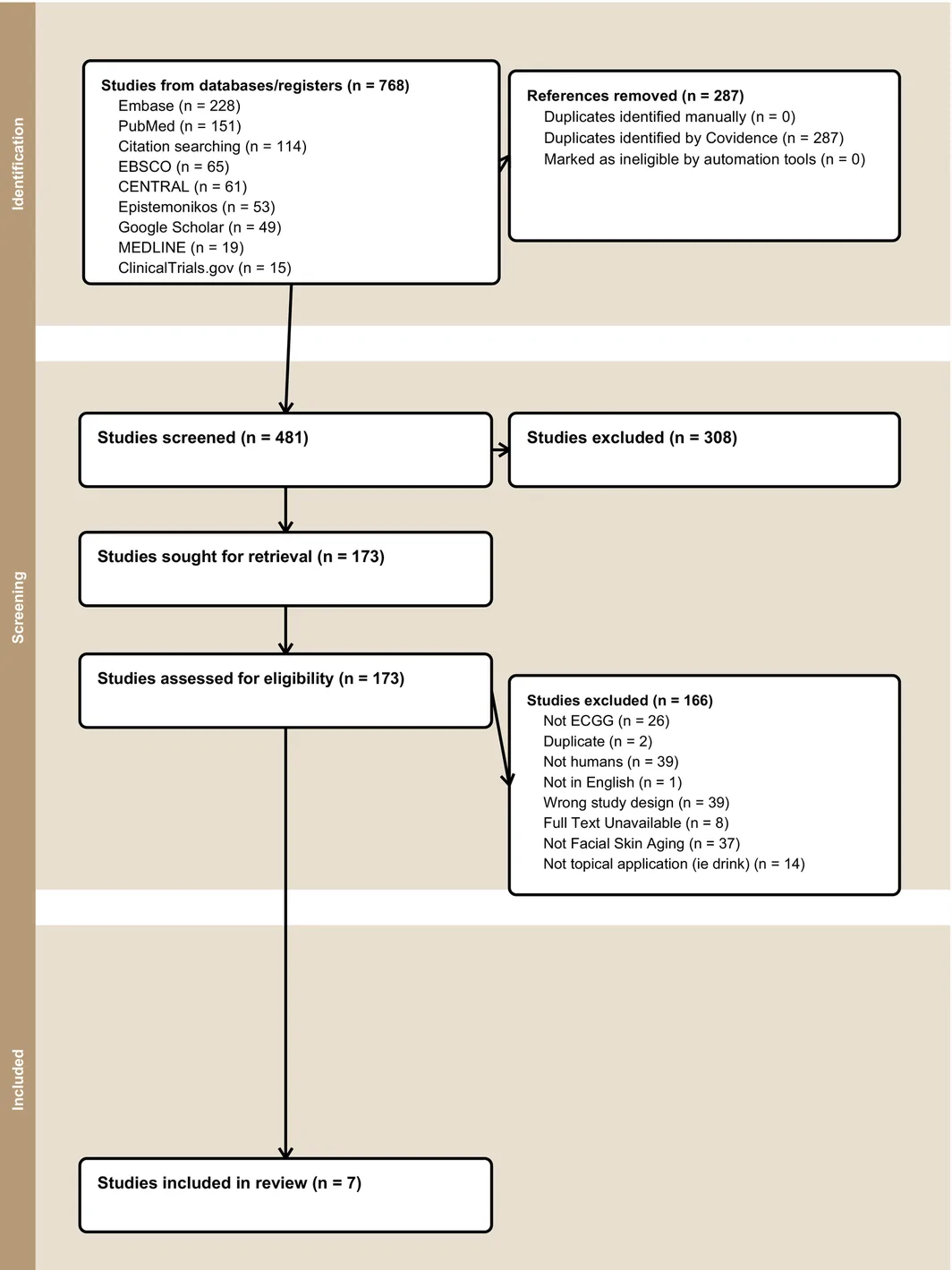
PRISMA diagram depicting the screening process that took place for this systematic review.

This systematic review is reported in accordance with the Preferred Reporting Items for Systematic Reviews and Meta‐Analyses (PRISMA) 2020 guidelines [9]. The PRISMA diagram can be found in Figure 1 [9]. The research question for the present review was formulated using the PICO model—Population (P): patients with visible facial aging and/or rhytids; Intervention (I): topical application of EGCG or GTE containing EGCG; Comparison (C): no adjuvant treatment or other adjuvant topical treatments; Outcomes (O): improvement in facial appearance as reflected by visible appearance in facial aging, improved elastic properties of skin, and reduction in rhytids.

### 2.2 Eligibility Criteria

The inclusion criteria required studies be: published in English, utilize topical EGCG and/or GTE containing EGCG, involved human patients, and evaluated the desired intervention. Due to the limited number of randomized controlled trials, studies of any design were included if they met all other criteria. Studies were excluded if they: did not have a full text available, did not include human patients (solely in vitro or animal studies), were duplicate articles, utilized ECGC and/or GTE non‐topically, were not focused on the face, or did not evaluate facial aging. The eligibility criteria were developed by two reviewers along with a librarian. The review team consisted of two medical students and one Otolaryngology‐Head & Neck Surgery resident physician.

### 2.3 Databases, Search Strategy, and Selection Process

One reviewer collaborated with a librarian to develop a comprehensive search strategy that utilized keywords and database‐specific controlled vocabulary (e.g., MeSH and Emtree terms) related to EGCG, facial aging, and outcomes. The initial search was performed on 6/26/24. Supplemental searches were completed on 8/29/24, 12/10/24, and 7/20/25. Searches were not restricted by language, publication date, or other limits. In addition to searching the electronic databases, reference lists of included studies were manually reviewed. Full database searches are available in Table S1.

Results were exported and uploaded into an online screening platform (Covidence) for deduplication and blinded independent assessment by two reviewers. First, titles and abstracts were screened followed by full‐text review using the predetermined criteria outlined above. Disagreements were resolved through a third independent reviewer.

### 2.4 Data Extraction and Synthesis

Data was recorded utilizing the pre‐formed template within the online screening platform by two independent reviewers. The main information extracted from each study included: publication information, inclusion/exclusion criteria, study demographics, EGCG intervention, other adjuvant treatments used, patient demographics, side effects, and outcomes.

Outcome measures and side effects were compared between studies. However, due to the small number of studies and heterogeneity in outcome measures, side effects, and study populations, a meta‐analysis could not be performed.

### 2.5 Risk of Bias in Individual Studies

The studies included in this systematic review were assessed for confounding factors, limitations, and biases using the quality assessment form found on the online screening platform. After data extraction, two independent reviewers completed the tool and differences were resolved by a third independent reviewer. The results are summarized in Figure S1 [10].

## 3 Results

### 3.1 Study Designs

Four hundred eighty‐one studies were identified for title and abstract screening, from which 173 made it to full‐text review, and seven were finally included for extraction [11, 12, 13, 14, 15, 16, 17]. A summary of study characteristics can be found in Table 1.

**TABLE 1:** A summation of the unique demographic characteristics of each individual study that was chosen for review.

Study	Study type	GTE preparation	Adjuvant therapy	Frequency and duration	Subjects	Withdrawals
Chiu 2005 [12]	Randomized controlled trial	10% GTE with 38% EGCG	300 mg green tea oral supplement	Twice a day for 8 weeks	40 women	3 for irritation and 1 for wrong application site
Sommer 2009 [17]	Case study	GTE (3 g per 250 mL water)	4 J/cm2 dermal dose of red light	Once a day for 4 weeks	1 woman	0
Domingo 2010 [16]	Split face study	2.5% w/w of EGCG in a silicone water emulsion	N/A	Twice a day for 6 weeks	3 women and 1 man ages 40–59	0
Mahmood 2011 [14]	Split face study	3% GTE in an oil emulsion	N/A	Once before bed for 8 weeks	10 men ages 25–40	0
Hong 2013 [15]	Randomized controlled trial	5% GTE	Tannase	Twice a day for 8 weeks	42 women ages 30–59	Not specified
Jagdeo 2021 [13]	Prospective cohort study	GTE cream	Vitamins C and E	Twice a day for 12 weeks	31 women ages 21–65	1 for unknown reason
Wisuitiprot 2022 [11]	Split face randomized controlled trial	1% (w/w) GTE with 0.006% EGCG (w/w) and 0.018% total catechins (w/w) on chitosan microparticle	N/A	Once before bed for 8 weeks	29 women ages 30–60	0

Three studies had a split face design, with each patient using themselves as a control [11, 14, 16]. One study had only one subject [17]. The last three used a control group [12, 13, 15]. Only two experiments included male subjects while the other five focused on women with visible photoaging [11, 12, 13, 14, 15, 16, 17].

The preparation of the topical GTE varied greatly, with many studies not including any information on how the GTE was prepared [12, 13, 15]. Of the studies that did provide this data, one used a chitosan microparticle cream, and the others used a variation of a water or oil emulsion [11, 14, 16, 17]. Each study had topical GTE applied once or twice daily for a minimum of 4 weeks [11, 12, 13, 14, 15, 16, 17]. Additionally, four of the studies also investigated how GTE works in combination with other substances or therapies; all of which found synergistic effects with their chosen adjuvant therapy [12, 13, 15, 17].

### 3.2 Measurement Methods

Very different measurements and scales were used to evaluate study outcomes. Three studies used self‐assessments, with two of these also utilizing a dermatologist assessment of the skin [12, 13, 15]. Two used skin punch biopsies with histological review to observe vascularity, collagen, and elastin contents of the treated skin [12, 16]. Histological review is an excellent way to monitor skin changes before and after treatment, but was not done in most of the trials because people are reluctant to have a facial biopsy.

A Cutometer, used as a less invasive alternative to biopsy, measures skin elasticity by applying negative pressure through a suction probe without removing skin [11, 14, 18]. Two studies also utilized a Corneometer which produces an electric current to measure the water content of the stratum corneum [11, 14, 19]. A Visioscan utilized LED UV‐A to observe the skin topography of the patients [11, 15, 17, 20]. A Mexameter measured melanin content and hyperpigmentation by emitting specific wavelengths and measuring refracted light [21]. The Tewameter measured transepidermal water loss by looking at water density changes [22]. Erythema was also assessed by one study using a Chromometer, as it can distinguish different shades of red [16].

### 3.3 Major Outcomes

#### 3.3.1 Wrinkles

GTE's effect on wrinkles was the most studied outcome with five trials investigating this outcome [11, 12, 13, 15, 17]. Using skin topography instruments, physician assessment, and patient self‐assessments, four of the five studies reported a decrease in fine line wrinkles with one also reporting improvement in deep wrinkles [11, 13, 15, 17]. These trials saw significantly diminished wrinkles at 2, 4, and 8 weeks. Only one study found no significant changes, but the changes in facial roughness and wrinkles in this study approached significance, suggesting that 8 weeks was not long enough [12]. A summary of study outcomes can be found in Table 2.

**TABLE 2:** An overview of the main outcomes of this systematic review.

Outcomes	Measurements	Positive (+), Negative (−), or No change (o)
**Chiu 2005**		
Wrinkles	Self assessment Dermatologist assessment	o o
Elasticity	Histological grading of skin biopsy	+
Dryness	Self assessment Dermatologist assessment	– o
Hyperpigmentation	Dermatologist assessment	o
**Sommer 2009**		
Wrinkles	Photographs	+
**Domingo 2010**		
Erythema	Chromometer	o
**Mahmood 2011**		
Elasticity	Cutometer	+
Water retention	Cutometer	+
**Hong 2013**		
Wrinkles	Visiometer Self assessment	+ +
**Jagdeo 2021**		
Wrinkles	Self assessment Dermatologist assessment	+ +
Elasticity	Self assessment	+
Erythema	Self assessment	o
Hyperpigmentation	Self assessment Dermatologist assessment (modified Griffiths 10‐point scale)	+ +
**Wisuitiprot 2022**		
Wrinkles	Visioscan	+
Elasticity	Cutometer	+
Erythema	Mexameter	o
Skin dullness (melanin index)	Mexameter	+
Water retention	Tewameter	o

*Note:* Endpoints, type of measurements, and clinical associations are provided for each study that was chosen for review.

#### 3.3.2 Elasticity

Skin elasticity was measured in four studies which all found some increase in elasticity with GTE use [11, 12, 13, 14]. One showed a significant increase in gross, net, and total elasticity at 4 weeks while another showed a significant increase in total elasticity at 1 week [11, 14]. A third showed an increase in self‐assessment scores of overall elasticity [13]. The final study utilized histology of GTE treated punch biopsies and found significant improvement in the amount of elastic tissue present [12].

#### 3.3.3 Pigmentation

Three trials looked at how GTE affects skin pigmentation [11, 12, 13]. One found a significant decrease in melanin at 6 weeks, and another found a significant decrease at 8 weeks based on patients' self‐assessment [11, 13]. The last study approached but did not reach significance by 8 weeks [12].

#### 3.3.4 Water Retention

The last outcome observed was water retention with two studies investigating this outcome. The first reported an increase in water retention at 3 weeks after GTE treatment [14]. The second found a significant increase in water retention from baseline, but there was no significance when compared to placebo treated skin [11].

#### 3.3.5 Other Outcomes

Other notable effects not noted in Table 2 include an improvement in radiance, firmness, scavenging ability, and density. Radiance increased significantly at 4 weeks and firmness increased significantly at 8 weeks [12]. Another study found scavenging ability significantly increased at 8 weeks [15]. Using ultrasound measurements, skin density was found to have increased 12 weeks in [13]. Self‐assessments completed by patients also showed an increase in confidence and self‐esteem after using GTE [13].

### 3.4 Side Effects

Five of the seven studies looked at possible side effects of topical GTE use on the face [11, 12, 13, 16] Erythema, inflammation, and dryness were used as measures of skin irritability. Three trials found no irritation according to these measures [11, 13, 15]. One study even found an improvement in skin inflammation and redness [16]. This improvement came from GTE decreasing VEGF and HIF‐1a, key regulators in angiogenesis [16].

However, one of the trials had conflicting results concerning the safety of GTE [12]. In this trial the dermatologist found no change in irritation but the patients reported high self‐assessment scores of irritation [12]. They reported that the GTE dried out their skin, with three patients even withdrawing [12]. In the discussion of this trial, the researchers addressed the self‐reported irritability and concluded that the dose of GTE was too high [12]. This is plausible since the dose in this study was up to ten times higher than the doses used in the other six studies [11, 12, 13, 14, 15, 16, 17].

## 4 Discussion

This systematic review suggests that topical GTE is a promising anti‐aging candidate for photoaged facial skin with minuscule safety concerns. All seven trials found a significant improvement in at least one outcome after using GTE. The most noticeable outcomes included improvements in wrinkles, elasticity, hyperpigmentation, and water retention of the skin. The most profound improvements were with wrinkles and elasticity.

Several studies have shown that eyes are the most important beauty factor of the face, as they preserve youthfulness [23, 24, 25]. This is the motivation for many of these studies testing on periorbital wrinkles. This area was also chosen because it is minimally affected by muscle contraction [11]. Most of these studies found that GTE helped diminish wrinkles, which coincides with its antioxidant effects [2, 3, 4, 5, 8, 11, 13, 15, 17]. Free radicals cause wrinkles by severely damaging skin cells and their DNA [11]. Since GTE has proven itself as a powerful free radical scavenger, it explains why wrinkle diminishment was prominent after several weeks of GTE [11]. Because only one of the studies found an improvement in deep‐set wrinkles, it prompts the question whether GTE could be more beneficial as a prophylactic agent.

Another main feature of aged skin is a loss of skin elasticity and collagen [14]. Elastic fiber and collagen degradation is also caused by free radical damage [3, 5]. The four studies that measured changes in elastic and collagen fibers all found some increase in these levels [11, 12, 13, 14]. One hypothesis for this outcome is that a topical application of GTE increases moisture content in the skin, contributing to more elastic fiber production [11]. Another factor could be due to GTE stabilizing collagen and preventing its degradation [11].

Studies have shown that catechins play a role in skin lightening, which is seen in two of these trials [11, 13]. Green tea catechins competitively inhibit the tyrosinase enzyme which is responsible for melanin biosynthesis [26, 27, 28, 29]. This is important because dark areas of skin are markers of photodamaged skin [14]. Skin lightening is directly correlated with a decrease in dullness and more radiant skin [11]. GTE shows potential as a routine skin lightener.

Water retention in skin is important because it builds up and protects the components of the ECM. The increase in water decreases viscosity of the ECM, allowing for less friction and softer skin [14]. This produces skin that is more elastic and radiant. One study showed an increase in water retention and another study almost approached significance. This hydrating effect demonstrates that GTE can potentially be used as a skin moisturizer.

Only one trial saw side effects with GTE use, but it was determined that the dosage was too high [12]. This is clinically relevant so that patients can be counseled on the risks of using high doses of GTE. A separate study found that although there was a decrease in angiogenic factors, there was no change in erythema levels [16]. While these decreased factors were not clinically relevant in this study, the researchers hypothesized that GTE could be used preventatively for erythema and photoaging [16].

This systematic review is limited by the small number of studies and the heterogeneity of measured outcomes. Ideally, most trials would have used the same instruments or questionnaires to measure the results. With more trials and more consistency in study designs, a meta‐analysis could be completed, and more conclusions could be drawn.

Several different adjuvant therapies were used, which is another limitation that made it difficult to draw conclusions as it is impossible to tell which component is responsible for the outcomes. One trial supplemented with 300 mg oral GTE and found inconclusive evidence on its efficacy [12]. Other trials found synergistic results with Vitamins C and E, tannase, and red light therapy [13, 15, 17]. More research needs to be done on each of these combination therapies with emphasis on what exact properties each individual component holds.

While these adjuvant therapy studies have limited interpretability for GTE alone as an anti‐aging product, three of the studies had no adjuvant therapy [11, 13, 14]. Analyzing these three studies together, one of which is a randomized controlled trial, more conclusions can be drawn on GTE as a standalone agent [11, 13, 14]. Elasticity was measured by two of these studies, both finding significant improvement in skin elasticity after topical GTE use, suggesting that increased elasticity can possibly be attributed to GTE alone [11, 14]. Two of these studies look at erythema and both found no significant difference, which is consistent with the adjuvant studies [11, 13]. Water retention was also evaluated by two of the studies, one finding an increase in retention, while the randomized controlled trial found no change [11, 14]. An improvement in wrinkles and skin dullness was also seen in the randomized controlled trial, but replication of these results would be needed to draw a strong conclusion about these outcomes.

Another limitation is the discrepancy in the frequency and length of treatment. The time periods vary between 4 and 12 weeks. There was no direct association seen between length of treatment and more significant results. The dosing and frequency of GTE use also varied, with no direct correlations to results. To provide physicians with accurate information on GTE, more clinical trials are needed to establish dosing, frequency, and duration.

Only two studies included men as subjects, and there was limited discussion about how GTE affects facial aging of men [14, 16]. There are many gender‐based differences of skin that are relevant to skin aging. Typically, men have thicker skin with more sebum production, which correlates with increased elasticity and waterproofing properties [30]. While these factors would be protective against skin aging, men also have higher cortisol levels putting them at a much higher risk of UV‐induced skin damage [30]. Furthermore, estrogen plays a key role in maintaining collagen density, skin thickness, and hydration, meaning postmenopausal women who experience a decline in estrogen may respond differently to topical GTE than premenopausal women [30]. Based on these differences, the results of this review would likely only be generalizable for women, which is another limitation of the study. One hypothesis for the major focus on women in these trials is that the main outcomes are focused on decreasing facial aging, which is associated with the beauty industry, which predominantly, but not exclusively, is marketed towards women. Future studies done on topical GTE should include more male subjects to fully understand the efficacy and side effects.

## 5 Conclusion

GTE has been proven to have significant antioxidant activity, but there is little research on whether this is synonymous with having anti‐aging properties. Despite the small number of published studies and lack of consistency in measurements, GTE with EGCG shows promise as an anti‐aging cosmeceutical with each of the seven studies finding improvement in at least one outcome. It also fulfills consumer demand as a natural product that would be minimally invasive. Further investigation on comparing topical GTE with EGCG to placebo cream on a larger population is needed. Additionally, a standardized set of measurements should be used to improve reproducibility. New research would help physicians better understand the properties of GTE so that they could accurately inform their patients on the benefits and risks.

## Author Contributions

**Taryn Lubbers:** conceptualization, project administration, data curation, methodology, investigation, formal analysis, and writing – original draft. **Cyrus W. Abrahamson:** data curation, methodology, investigation, formal analysis, and writing – original draft. **Paavali A. Hannikainen:** investigation, formal analysis, and writing – review and editing. **Daniel Stuart:** data curation, methodology, resources, and writing – review and editing. **James C. Wang:** supervision and writing – review and editing.

## Ethics Statement

The authors confirm that the ethical policies of the journal, as noted on the journal's author guidelines page, have been adhered to. Approval from an institutional ethical review board was not required for this study as it is a systematic review of publicly available literature. The authors declare no conflicts of interest. The authors received no financial support for the research or publication of this article.

## Consent

The authors have nothing to report.

## Conflicts of Interest

The authors declare no conflicts of interest.

## Supporting information


**Figure S1:** Risk‐of‐bias assessment for each included study, completed independently by two reviewers with disagreements resolved by a third reviewer.

**Table S1:** List of all databases searched with their corresponding reproducible queries as well as all supplemental searches that were completed.

## Data Availability

Data sharing not applicable to this article as no datasets were generated or analyzed during the current study.
