# Supplementary material for: Efficacy and Side Effects of Green Tea Extract on Facial Skin Aging: A Systematic Review
Source: J Cosmet Dermatol. 2026 Aug 28;25(9):e71125. doi: 10.1111/jocd.71125 (PMC13523624; doi:10.1111/jocd.71125)
Supplement: Supplementary file 1 [file JOCD-25-e71125-s001.docx]

**Figure S1**

Risk-of-bias assessment for each included study, completed independently by two reviewers with disagreements resolved by a third reviewer.
