# Supplementary material for: Efficacy and Side Effects of Green Tea Extract on Facial Skin Aging: A Systematic Review
Source: J Cosmet Dermatol. 2026 Aug 28;25(9):e71125. doi: 10.1111/jocd.71125 (PMC13523624; doi:10.1111/jocd.71125)
Supplement: Supplementary file 2 [file JOCD-25-e71125-s002.docx]

**Table S1**

| **Primary Searches** | |
| --- | --- |
| **Database** | **Reproducible Search** |
| Pubmed | (((face[tiab]) OR (facial[tiab])) AND ("green tea"[tiab])) OR ((((((green tea extract skin aging) OR ((((((((((green tea extract) OR (veregren)) OR (exolise)) OR (green tea catechin)) OR (green tea extract polyphenone)) OR (green tea extract ar25)) OR ("green tea"[tiab])) OR (sinecatechins)) OR (("epigallocatechin gallate") OR ("egcg"[tiab]))) AND ((((((((photoaging) OR ("anti-aging")) OR (ageing)) OR (wrinkle[tiab])) OR (wrinkles[tiab])) OR ("facial skin")) AND ((((((((face) OR (facial)) OR (cheek)) OR (chin)) OR (eyelids)) OR (eyebrows)) OR (nose)) OR (forehead))) OR (((((skin aging) OR (facial wrinkles)) OR (wrinkling)) OR (("Skin Aging"[Mesh]) AND "prevention and control" [Subheading])) AND ((((((((face) OR (facial)) OR (cheek)) OR (chin)) OR (eyelids)) OR (eyebrows)) OR (nose)) OR (forehead)))))) OR ((skin aging green tea) AND (Therapy/Narrow[filter]))) OR (((((((((((((green tea extract) OR (verfahren)) OR (veregren)) OR (exolise)) OR (green tea catechin)) OR ("green tea"[tiab])) OR ("green tea extract AR25" [Supplementary Concept] OR "green tea extract polyphenone E" [Supplementary Concept])) AND ((((((((face) OR (facial)) OR (cheek)) OR (chin)) OR (eyebrow)) OR (eyelid)) OR (nose)) OR (forehead))) AND ((facial[tiab]) OR (face[tiab]))) OR ((green tea extract face) AND (Therapy/Broad[filter]))) AND ((facial[tiab]) OR (face[tiab]))) AND ("green tea"[tiab])) OR (green tea wrinkles face))) AND ((face[tiab]) OR (facial[tiab]))) OR (((((((((((((green tea extract) OR (verfahren)) OR (veregren)) OR (exolise)) OR (green tea catechin)) OR ("green tea"[tiab])) OR ("green tea extract AR25" [Supplementary Concept] OR "green tea extract polyphenone E" [Supplementary Concept])) AND ((((((((face) OR (facial)) OR (cheek)) OR (chin)) OR (eyebrow)) OR (eyelid)) OR (nose)) OR (forehead))) AND ((facial[tiab]) OR (face[tiab]))) OR ((green tea extract face) AND (Therapy/Broad[filter]))) AND ((facial[tiab]) OR (face[tiab]))) AND ("green tea"[tiab])) OR (green tea wrinkles face))) |
| Ovid MEDLINE | 1: ("green tea extract" or exolise or "green tea catechin" or "green tea extract polyphenone" or "green tea extract ar25").af. or "green tea".ti. or "green tea".ab. or sinecatechins.af. or "epigallocatechin gallate".af. or "egcg".af. or veregren.af.  2: ("skin aging" or "facial wrinkles" or wrinkling or photoaging or ageing).af. or wrinkle.ti. or wrinkle.ab. or "facial skin".af. or "anti-aging".af.  3: face.sh. or facial.af. or cheek.af. or chin.af. or eyelids.af. or face.ti. or face.ab. or eyebrows.af. or nose.af. or forehead.af.  4: 1 and 2 and 3  5: green tea.mp.  6: skin aging.mp. or exp Skin Aging/  7: wrinkles.mp.  8: 6 or 7  9: 5 and 8  10: 4 or 9  11: limit 10 to humans |
| CENTRAL | 1: ("green tea extract" or exolise or "green tea catechin" or "green tea extract polyphenone" or "green tea extract ar25").af. or "green tea".ti. or "green tea".ab. or sinecatechins.af. or "epigallocatechin gallate".af. or "egcg".af. or veregren.af.  2: ("skin aging" or "facial wrinkles" or wrinkling or photoaging or ageing).af. or wrinkle.ti. or wrinkle.ab. or "facial skin".af. or "anti-aging".af.  3: face.sh. or facial.af. or cheek.af. or chin.af. or eyelids.af. or face.ti. or face.ab. or eyebrows.af. or nose.af. or forehead.af.  4: 1 and 2 and 3  5: green tea.mp.  6: skin aging.mp. or exp Skin Aging/  7: wrinkles.mp.  8: 6 or 7  9: 5 and 8  10: 4 or 9  11: limit 10 to humans |
| Embase | (('green tea extract'/exp OR 'green tea extract' OR (('green'/exp OR green) AND ('tea'/exp OR tea) AND ('extract'/exp OR extract)) OR 'green tea':ab,ti OR veregren OR 'exolise'/exp OR exolise OR 'green tea catechin'/exp OR 'green tea catechin' OR 'green tea polyphenol'/exp OR 'green tea polyphenol' OR 'green tea polyphenone' OR (('green'/exp OR green) AND ('tea'/exp OR tea) AND polyphenone) OR 'sinecatechins'/exp OR sinecatechins OR 'epigallocatechin gallate'/exp OR 'epigallocatechin gallate' OR 'egcg'/exp OR 'egcg') AND ('skin aging'/exp OR 'skin aging' OR (('skin'/exp OR skin) AND ('aging'/exp OR aging)) OR wrinkles:ab,ti OR 'wrinkles'/exp OR wrinkles OR 'wrinkle'/exp OR 'wrinkling'/exp OR wrinkling OR 'photoaging'/exp OR photoaging OR 'anti-aging' OR 'ageing'/exp OR ageing OR 'facial skin'/exp OR 'facial skin' OR 'cutaneous parameters'/exp) AND ('face'/exp OR face OR 'chin'/exp OR chin OR facial OR 'cheek'/exp OR cheek OR 'eyelids'/exp OR eyelids OR 'eyebrows'/exp OR 'nose'/exp OR nose OR 'forehead'/exp OR forehead) OR (('cutaneous parameters'/exp OR 'cutaneous parameters') AND 'prevention and control'/exp AND 'sinecatechins'/exp) OR 'green tea skin aging' OR (('green'/exp OR green) AND ('tea'/exp OR tea) AND ('skin'/exp OR skin) AND ('aging'/exp OR aging)) OR (('adult'/exp OR 'adult' OR 'adults' OR 'grown-ups' OR 'grownup' OR 'grownups') AND ('human'/exp OR 'homo sapiens' OR 'human' OR 'human being' OR 'human body' OR 'human race' OR 'human subject' OR 'humans' OR 'man (homo sapiens)') AND ('sinecatechins'/exp OR 'camellia chinensis extract' OR 'camellia sinensis extract' OR 'green tea extract' OR 'sinecatechins' OR 'veregen' OR 'green tea') AND ('cutaneous parameters'/exp OR 'aging skin' OR 'cutaneous parameters' OR 'cutis senilis' OR 'senile skin' OR 'skin aging'))) AND ('article'/it OR 'review'/it) AND [humans]/lim AND ([adult]/lim OR [aged]/lim OR [very elderly]/lim) |
| **Supplemental Searches/Journal Searches** | |
| **Database/Journal** | **Reproducible Search** |
| Journal of Drugs in Dermatology | Green tea |
| Clinics in Dermatology | Green tea and green tea aging |
| Google Scholar | Green tea skin aging aging OR wrinkles "green tea" |
| Epistemonikos | Green tea skin aging |
| EBSCO Discovery | (TI "green tea" OR AB "green tea" OR TX "green tea extract") AND (skin aging) |
| International Clinical Trials Registry Platform (ICTRP) | Green tea |
| ClinicalTrials.gov | Green tea and green tea aging |

List of all databases searched with their corresponding reproducible queries as well as all supplemental searches that were completed.
